# Associations of alanine aminotransferase/aspartate aminotransferase with insulin resistance and β-cell function in women

**DOI:** 10.1038/s41598-023-35001-1

**Published:** 2023-05-15

**Authors:** Satomi Minato-Inokawa, Ayaka Tsuboi-Kaji, Mari Honda, Mika Takeuchi, Kaori Kitaoka, Miki Kurata, Bin Wu, Tsutomu Kazumi, Keisuke Fukuo

**Affiliations:** 1 Research Institute for Nutrition Sciences, Mukogawa Women’s University, 6-46, Ikebiraki-cho, Nishinomiya, Hyogo 663-8558 Japan; 2 Laboratory of Community Health and Nutrition, Department of Bioscience, Graduate School of Agriculture, Ehime University, Matsuyama, Ehime Japan; 3 Department of Pharmacoepidemiology, Graduate School of Medicine and Public Health, Kyoto University, Kyoto, Japan; 4 Department of Nutrition, Osaka City Juso Hospital, Osaka, Japan; 5 Open Research Center for Studying of Lifestyle-Related Diseases, Mukogawa Women’s University, Nishinomiya, Hyogo Japan; 6 Department of Health, Sports, and Nutrition, Faculty of Health and Welfare, Kobe Women’s University, Kobe, Hyogo Japan; 7 Department of Advanced Epidemiology, Noncommunicable Disease (NCD) Epidemiology Research Center, Shiga University of Medical Science, Otsu, Shiga Japan; 8 Department of Food Sciences and Nutrition, Mukogawa Women’s University, Nishinomiya, Hyogo Japan; 9 Department of Endocrinology, First Affiliated Hospital of Kunming Medical University, Kunming, Yunnan China; 10 Department of Medicine, Kohan Kakogawa Hospital, Kakogawa, Hyogo Japan

**Keywords:** Biomarkers, Medical research

## Abstract

We tested whether alanine aminotransferase/aspartate aminotransferase (ALT/AST), a marker of hepatosteatosis, associates with insulin resistance, β-cell function and postglucose glycemia. We studied 311 young and 148 middle-aged Japanese women, whose BMI averaged < 23.0 kg/m^2^. Insulinogenic index and Matsuda index were evaluated in 110 young and 65 middle-aged women. In two groups of women, ALT/AST was associated positively with homeostasis model assessment insulin resistance (HOMA-IR) and inversely with Matsuda index. In middle-aged women only, the ratio was also associated positively with fasting and postload glycemia and HbA1c. The ratio showed negative association with disposition index (a product of insulinogenic index and Matsuda index). On multivariate linear regression analysis, HOMA-IR emerged as a single determinant of ALT/AST in young and middle-aged women (standardized β: 0.209, p = 0.003 and 0.372, p = 0.002, respectively). ALT/AST was associated with insulin resistance and β-cell function even in non-obese Japanese women, suggesting a pathophysiologic basis in its prediction of diabetic risk.

## Introduction

Epidemiologic studies^1–4^ have shown that modest elevations of serum liver enzymes, such as alanine aminotransferase (ALT) and aspartate aminotransferase (AST), are associated with an increased risk of subsequently developing type 2 diabetes, which are characterized by insulin resistance and impaired β-cell function. The aminotransferases, particularly ALT, are markers of nonalcoholic fatty liver disease (NAFLD), which are also characterized by insulin resistance^5,6^. ALT/AST can be used to evaluate the degree of hepatic fat infiltration and hepatic steatosis^7–11^. For example, in a Framingham study, it was shown that ALT/AST could identify hepatic steatosis more accurately than using ALT or AST alone^9^. A recent population-based longitudinal study has revealed that the increase in the ALT/AST ratio was closely associated with the risk of new-onset NAFLD in nonobese Chinese individuals^12^.

Although insulin resistance and impaired β-cell function are hallmarks of type 2 diabetes, studies confirm more severe functional insulin secretory defects in lean individuals compared to the obese phenotype in some Asian countries^13^. We^14^ demonstrated that middle-aged Japanese people with prediabetes had reduced glucose-induced insulin secretion and higher circulating orosomucoid, an acute-phase glycoprotein produced mainly in the liver^15^, in the absence of insulin resistance. However, studies which assessed β-cell function and postglucose glycemia in relation to serum concentrations of liver enzymes are limited as discussed later. We, therefore, investigated whether ALT/AST may be associated with β-cell function and postglucose glycemia in addition to insulin resistance. As described in detail in “Methods”, glucose-induced insulin secretion was evaluated by insulinogenic index (IGI), insulin resistance-corrected insulin secretion (β-cell function) by the oral disposition index (ODI) and insulin resistance/sensitivity by homeostasis model assessment (HOMA-IR) and Matsuda index. These analyses were done in young and middle-aged Japanese women, populations in which confounding factors are so scarce^16–18^. Because liver fat accumulation has been reported to be associated with increased serum orosomucoid in addition to branched chain and aromatic amino acids^19^, and because orosomucoid and C-reactive protein (CRP) were associated with an increased risk of both type 2 diabetes and cardiovascular disease^20^, we also studied whether ALT/AST may be associated with serum orosomucoid. Lastly, we examined whether ALT/AST may be associated these variables stronger than ALT alone.

## Methods

We examined 311 young and 148 middle-aged women as previously reported^16–18^. Among 148 middle-aged women, 137 (92.6%) reported to have regular menstrual cycles (premenopausal). The present study was done between 2004 and 2007 and these women participated as volunteers as previously reported in detail^16^. Young women were female Japanese students of Department of Food Sciences and Nutrition, Mukogawa Women's University and middle-aged women were their biological mothers of 148 students who participated in the study. Subjects were excluded from the study when they reported to have clinically diagnosed acute or chronic inflammatory diseases, endocrine, cardiovascular, hepatic, renal diseases, hormonal contraception, unusual dietary habits. This research followed the tenets of the Declaration of Helsinki. All participants gave written informed consent after the experimental procedure had been explained.

Blood samples were obtained in the morning after 12-h overnight fast. Oral glucose tolerance test (OGTT) was performed with 75-g glucose administration in 118 female students and 65 mothers. Blood samples were taken at min 0 (fasting), 30, 60 and 120 for glucose and insulin analysis. Plasma glucose was determined by the hexokinase/glucose-6-phosphate dehydrogenase method [interassay coefficient of variation (CV) < 2%]. Serum insulin was measured by an ELISA method with a narrow specificity excluding des-31, des-32, and intact proinsulin (interassay CV < 6%). Insulin resistance/sensitivity was determined by HOMA-IR using fasting plasma glucose and insulin levels^21^ and Matsuda index using glucose and insulin levels during OGTT^22^. IGI was calculated as incremental insulin concentrations (μU/mL) divided by incremental glucose concentrations (mg/dL) during the first 30 min of OGTT^23^. ODI was calculated as the product of IGI and Matsuda index. Area under the glucose and insulin concentration curve during OGTT (AUCg and AUCi, respectively) was calculated using trapezoidal method. Prediabetes and diabetes were diagnosed based on glycemia criteria (fasting and 2-h glucose concentrations) of the American Diabetes Association^24^.

Serum liver enzymes were measured using an autoanalyzer (AU5232, Olympus, Tokyo, Japan). Highly-sensitivity C-reactive protein (hsCRP) concentrations were measured by an immunoturbidimetric assay with the use of reagents and calibrators from Dade Behring Marburg GmbH (Marburg, Germany, with an interassay CV less than 5%). Orosomucoid concentrations were measured by an immunoturbidimetric method using a commercially available kit (N Antiserum to Human α1-acid Glycoprotein, Siemens Healthcare Diagnostics, Tokyo, Japan) and an autoanalyzer (JCA-BM6010, JEOL, Tokyo, Japan). Intra-assay and interassay CV at 87 mg/dL was 1.4% and 1.7%, respectively.

Body weight, height and waist circumference were measured after an overnight fast with a light cloth and shoes off and BMI was calculated. Weight and height were measured to the nearest 0.1 kg and 0.1 cm, respectively. Whole-body dual-energy X-ray absorptiometry (DXA) (Hologic QDR-2000, software version 7.20D, Waltham, MA) was used to measure lean tissue mass, fat mass, and bone mineral mass for arms, lower-body, trunk and the total body^17^.

### Statistical analysis

Data were presented as mean ± SD unless otherwise stated. Due to deviation from normal distribution, ALT, IGI, ODI and hsCRP were logarithmically transformed for analyses. Bivariate correlations of ALT/AST with anthropometric and metabolic parameters were evaluated by Pearson’s correlation analysis. Stepwise multivariate linear regression analyses were performed to further identify the most significant variables contributing to the variation of ALT/AST. Comparisons between two groups were made with two-sample t-test. Differences among three groups were analyzed using analysis of variance and then Bonferroni's multiple comparison procedure. A two-tailed p < 0.05 was considered statistically significant. All calculations were performed with SPSS system 23 (SPSS Inc, Chicago, IL).

## Results

On average, middle-aged mothers and their daughters were nonobese rather slim and had normal mean serum ALT and AST (Table 1). Middle-aged mothers compared with their daughters had higher BMI, percentage body fat, waist circumference and trunk fat and hence higher HbA1c, serum ALT, AST and ALT/AST. However, their BMI, waist and ALT averaged 22 kg/m^2^, 79 cm and 20 U/L, respectively.

**Table 1:** Clinical features of young and middle-aged Japanese women.

OGTT	Young	Middle-aged	p values
	n = 307	n = 148	
	n = 118	n = 65	
Age (years)	20.5 ± 1.2	49.8 ± 3.6	< 0.001
BMI (kg/m^2^)	20.4 ± 2.2	22.0 ± 2.8	< 0.001
Waist (cm)	71.2 ± 5.7	78.7 ± 8.1	< 0.001
Percentage body fat (%)	27.8 ± 5.5	30.1 ± 7.3	0.001
Percentage trunk fat (%)	28.6 ± 6.6	32.9 ± 8.7	< 0.001
Fasting glucose (mg/dL)	83 ± 7	89 ± 14	< 0.001
30-min glucose (mg/dL)	122 ± 23	138 ± 31	< 0.001
1-h glucose(mg/dL)	104 ± 33	133 ± 42	< 0.001
2-h glucose (mg/dL)	94 ± 23	113 ± 28	< 0.001
Fasting insulin (μU/mL)	6.2 ± 3.4	5.4 ± 2.8	0.014
30-min insulin (μU/mL)	54 ± 34	39 ± 24	0.001
1-h insulin (μU/mL)	44 ± 27	42 ± 24	0.678
2-h insulin (μU/mL)	42 ± 25	37 ± 29	0.239
HbA1c (%)	5.2 ± 0.2	5.5 ± 0.4	< 0.001
HOMA-IR	1.28 ± .78	1.21 ± .71	0.354
IGI	3.8 ± 11.8	1.0 ± 1.0	0.012
Oral disposition index	39 ± 129	9 ± 10	0.015
Matsuda index	9.1 ± 4.2	9.5 ± 4.6	0.505
AUCg (mg/dL/2 h)	207 ± 42	248 ± 56	< 0.001
AUCi (μU/mL/2 h)	82 ± 39	71 ± 38	0.064
AST (U/L)	17 ± 5	21 ± 13	0.001
ALT (U/L)	13 ± 7	20 ± 18	< 0.001
ALT/AST	0.74 ± 0.24	0.91 ± 0.26	< 0.001
Orosomucoid (mg/dL)	125 ± 26	148 ± 35	< 0.001
hsCRP (μg/dL)	29 ± 69	17 ± 54	0.067

Mean ± SD. HOMA-IR: homeostasis model assessment-insulin resistance. IGI: insulinogenic index, AUCg and AUCi: area under the glucose and insulin concentration curve, respectively, AST and ALT: aspartate- and alanine-aminotransferase, respectively, hsCRP: high-sensitivity C-reactive protein.

OGTT revealed that prediabetes was found in 7 of 118 young women^25^ and 11 of 65 middle-aged women^14^ whereas none had diabetes. Middle-aged women had lower ODI and hence higher glycemia at four time points (Table 1). Matsuda index and HOMA-IR did not differ between two groups.

In young women, there was no significant association of log ALT with variables depicted in Table 1 (data not shown), including fasting insulin (r = 0.08, p = 0.15), HOMA-IR (r = 0.104, p = 0.06) and log HOMA-IR (r = 0.101, p = 0.07). In contrast, ALT/AST showed positive associations with fasting insulin, HOMA-IR and, AUCi and inversely with Matsuda index (Table 2). However, it showed no associations with percentage body fat, percentage trunk fat, and all glycemic variables studied although there was a weak association with BMI.

**Table 2:** Simple linear correlation analyses of alanine aminotransferase (ALT) and alanine aminotransferase/aspartate aminotransferase (ALT/AST) in young and middle-aged Japanese women.

	Young women		Middle-aged women			
	ALT/AST		log ALT		ALT/AST	
	r	p values	r	p values	r	p values
BMI	**0.117**	**0.041**	**0.175**	**0.034**	**0.325**	** < 0.001**
Waist	0.081	0.248	**0.263**	**0.001**	**0.366**	** < 0.001**
Percentage body fat	0.027	0.634	**0.230**	**0.006**	**0.345**	** < 0.001**
Percentage trunk fat	0.057	0.323	**0.275**	**0.001**	**0.374**	** < 0.001**
Fasting glucose	0.046	0.423	0.042	0.610	**0.259**	**0.001**
30-min glucose	0.121	0.193	**0.327**	**0.008**	**0.309**	**0.012**
1-h glucose	0.077	0.409	0.152	0.228	**0.288**	**0.020**
2-h glucose	0.050	0.592	0.229	0.066	**0.270**	**0.029**
Fasting insulin	**0.167**	**0.003**	**0.271**	**0.001**	**0.424**	** < 0.001**
30-min insulin	0.040	0.666	0.097	0.442	0.206	0.100
1-h insulin	**0.260**	**0.004**	0.073	0.565	**0.288**	**0.020**
2-h insulin	0.146	0.115	**0.268**	**0.031**	**0.431**	** < 0.001**
HbA1c	0.013	0.825	0.072	0.385	**0.194**	**0.018**
HOMA-IR	**0.163**	**0.004**	**0.248**	**0.002**	**0.456**	** < 0.001**
Matsuda index	**− 0.199**	**0.030**	− 0.081	0.519	**− 0.387**	**0.001**
AUCg	0.098	0.289	0.236	0.059	**0.323**	**0.009**
AUCi	**0.207**	**0.025**	0.171	0.172	**0.373**	**0.002**
log IGI	− 0.069	0.458	− 0.217	0.082	− 0.103	0.416
log ODI	− 0.162	0.079	**− 0.302**	**0.015**	**− 0.354**	**0.004**
Orosomucoid	0.053	0.549	**0.268**	**0.031**	**0.323**	**0.009**
log hsCRP	0.035	0.544	0.130	0.116	0.142	0.085

Data are correlation coefficients (r). Significant correlations are indicated by bold values. ODI: oral disposition index. Other abbreviations are the same as in Table 1.

**Figure 1:**
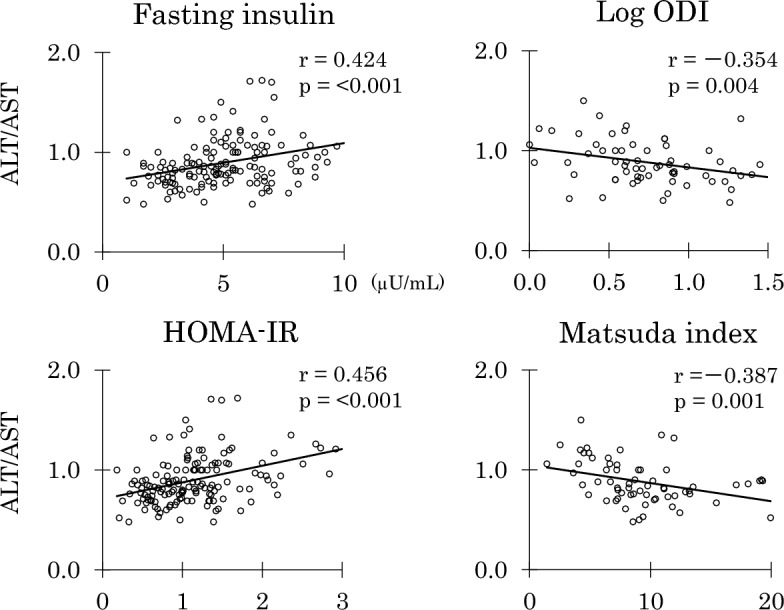
Correlations of alanine aminotransferase/aspartate aminotransferase (ALT/AST) with fasting insulin, homeostasis model assessment-insulin resistance (HOMA-IR), log oral disposition index (ODI), and Matsuda index in middle-aged Japanese women.

In middle-aged women, associations of ALT/AST were stronger than those of log ALT (Table 2). ALT/AST was positively associated with BMI, waist, percentage body and trunk fat. The ratio showed positive associations with all glycemic variables studied (glycemia at four time points, AUCg and HbA1c). In addition, the ratio showed significant associations with all markers of insulin resistance/insulin sensitivity studied (fasting and 2-h insulin, AUCi, HOMA-IR, and Matsuda index) (Fig. 1). Further, ALT/AST showed negative association with log ODI although it was not associated with IGI. Associations of ALT/AST with orosomucoid and log hsCRP were significant and tended to be significant, respectively, in middle-aged women.

We have done multivariate analysis for ALT/AST as a dependent variable. The model included HOMA-IR, Matsuda index, log ODI and AUCg as independent variables in young and middle-aged women. HOMA-IR emerged as a single determinant of ALT/AST in both young and middle-aged women (standardized β: 0.209, p = 0.003 and 0.372, p = 0.002, respectively). Including orosomucoid as an additional independent variable did not change the results in middle-aged women.

**Figure 2:**
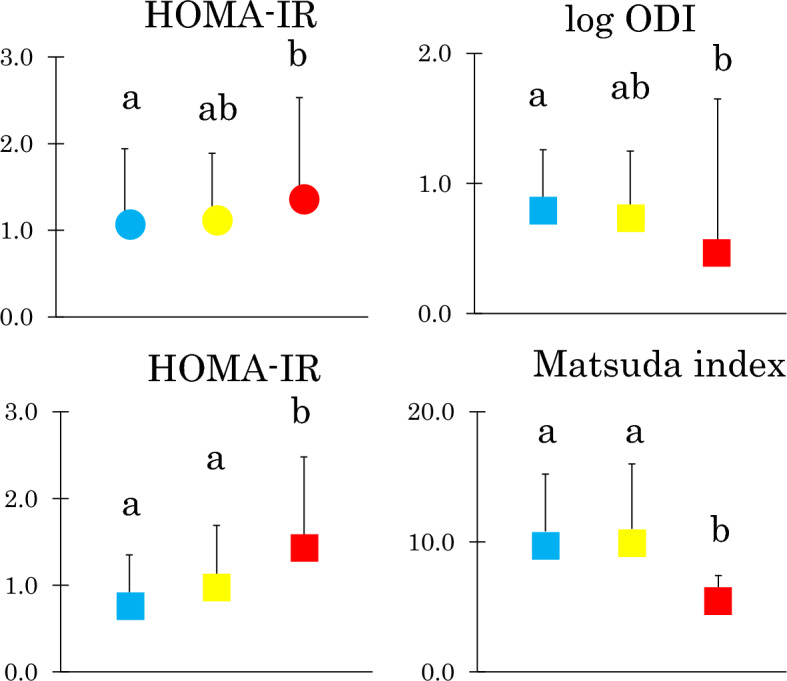
Homeostasis model assessment-insulin resistance (HOMA-IR), log oral disposition index (ODI) and Matsuda index in young (circles) and middle-aged Japanese women (squares) grouped according to tertile of alanine aminotransferase/aspartate aminotransferase (ALT/AST). Mean ± SD. Means not sharing common letter are significantly different with each other at p < 0.05 or less by Bonferroni’s multiple comparison procedure. Blue, yellow and red symbols represent the low, median and high tertile, respectively. P values: HOMA-IR: young: p = 0.04, middle-aged: p < 0.001 vs. low, p = 0.003 vs. median, log ODI: p = 0.01, Matsuda index: p = 0.004 vs. low, p = 0.003 vs. median.

Young and middle-aged Japanese women were grouped according to tertile of ALT/AST. HOMA-IR increased in two groups of women and log ODI decreased in middle-aged women in a stepwise fashion from the low through median to high tertile (Fig. 2). Matsuda index was lower in the high compared to low and median tertile in middle-aged women. In young women, differences in Masuda index and log ODI were not significant after Bonferroni’s multiple comparison procedure because associations were weak (Table 2).

## Discussion

The current study confirmed stronger associations of ALT/AST than those of log ALT and demonstrated an association of ALT/AST, a marker of hepatosteatosis^7–12^, with insulin resistance (higher HOMA-IR) in women in early adult life in addition to midlife. In addition, it was also associated with not only fasting and postglucose glycemia and HbA1c but also serum orosomucoid, a risk factor for type 2 diabetes^20^, in middle-aged women. Further, the ratio was associated with impaired β-cell function (lower ODI) and hence slower glucose disposal rate during OGTT (higher AUCg).

There is a strong relationship between fat accumulation in the liver and whole-body insulin resistance independent of visceral adiposity, a major regulator of both liver fat and insulin resistance^26^. An association of ALT/AST, a marker of hepatosteatosis^7–12^, with HOMA-IR, a robust tool for the assessment of insulin resistance, has been demonstrated in studies from Japan^27^, China^28^ and Korea^29^. The present study confirmed this association in middle-aged Japanese women and extended that this association was evident even in Japanese women in early adult life whose waist and ALT averaged 71 cm and 13 U/L, respectively, suggesting minimum abdominal and hepatic lipid accumulation, respectively.

Studies which assessed β-cell function and postglucose glycemia in relation to serum concentrations of liver enzymes are limited. We found a single study which studied associations with β-cell function. Pinnaduwage et al.^30^ evaluated the association of changes in liver enzymes with changes in insulin sensitivity, β-cell function and glycemia in 336 women with varying degrees of previous gestational glucose metabolic status at 1 and 3 years postpartum. They found that an increase in ALT/AST predicted lower β-cell function, worsening insulin sensitivity and higher fasting glucose at 3 years. In the present study, higher ALT/AST in middle-aged Japanese women was associated with higher insulin resistance and impaired β-cell function (lower ODI) and hence slower glucose disposal rate during OGTT (higher AUCg).

Song et al.^31^ studied 1128 pregnant women who underwent serum liver enzyme measurements and 75 g OGTT and assessed the relationship of liver enzymes and glycemia during OGTT. They found associations of ALT/AST with fasting and postglucose glycemia in a total of 1128 pregnant women, which were found in 65 middle-aged Japanese women in the present study. They also found that higher ALT/AST as well as triglyceride/high-density lipoprotein cholesterol, a lipid marker of insulin resistance^32^, was an independent risk factor of gestational diabetes.

An association of ALT/AST with HbA1c in the present study may be related to observations that the severity grades of steatosis evaluated by transient elastography or abdominal ultrasound correlated with HbA1C levels^33,34^. An association with orosomucoid in the present study may be related to a study^19^ which showed that already in its early stage liver fat accumulation was associated with increased serum orosomucoid and branched-chain amino, which may be associated with adipose tissue dysfunction. Hepatic orosomucoid expression has recently found to correlate with NAFLD parameters in obese mice with NAFLD induced by high-fat Western diet together with liquid sugar (fructose and sucrose) feeding^35^.

Associations were stronger in middle aged women than in young women. This may be related to the observation that associations among biomarkers of metabolic syndrome are stronger in obese women than in lean women^36^, as middle-aged women had greater percentage body fat, waist circumference and trunk fat than did younger women although middle-aged women were not obese.

The strength of this study includes homogeneous study population with scarce confounding factors^17,18^. Several limitations of this study include the cross-sectional design, relatively small sample size, and a single measurement of biochemical variables. We used many surrogates in the present study, which may be less accurate. Finally, as we studied Japanese women only, results may not be generalized to other sex, races or ethnicities.

In conclusions, ALT/AST, a marker of hepatosteatosis, was associated with insulin resistance, β-cell function and glucose disposal rate even in non-obese Japanese women, suggesting a pathophysiologic basis in its prediction of diabetic risk.

## Data Availability

The datasets used and/or analyzed during the current study available from the corresponding author on reasonable request.
